# Inter-vendor performance of deep learning in segmenting acute ischemic lesions on diffusion-weighted imaging: a multicenter study

**DOI:** 10.1038/s41598-021-91467-x

**Published:** 2021-06-14

**Authors:** Deniz Alis, Mert Yergin, Ceren Alis, Cagdas Topel, Ozan Asmakutlu, Omer Bagcilar, Yeseren Deniz Senli, Ahmet Ustundag, Vefa Salt, Sebahat Nacar Dogan, Murat Velioglu, Hakan Hatem Selcuk, Batuhan Kara, Ilkay Oksuz, Osman Kizilkilic, Ercan Karaarslan

**Affiliations:** 1 Department of Radiology, Acibadem Mehmet Ali Aydinlar University School of Medicine, Istanbul, Turkey; 2 Department of Software Engineering and Applied Sciences, Bahcesehir University, Istanbul, Turkey; 3 Cerrahpaşa Medical Faculty, Neurology Department, Istanbul University-Cerrahpasa, Istanbul, Turkey; 4 Department of Radiology, Istanbul Mehmet Akif Ersoy Thoracic and Cardiovascular Surgery Training and Research Hospital, Halkali/Istanbul, Turkey; 5 Radiology Department, Istanbul Silivri State Hospital, Istanbul, Turkey; 6 Cerrahpaşa Medical Faculty, Radiology Department, Istanbul University-Cerrahpasa, Istanbul, Turkey; 7 Radiology Department, Istanbul Gaziosmanpasa Training and Research Hospital, Istanbul, Turkey; 8 Radiology Department, Istanbul Fatih Sultan Mehmet Training and Research Hospital, Istanbul, Turkey; 9 Radiology Department, Istanbul Bakırköy Sadi Konuk Training and Research Hospital, Istanbul, Turkey; 10 Department of Software Engineering and Applied Sciences, Istanbul Technical University, Istanbul, Turkey

**Keywords:** Stroke, Brain imaging, Magnetic resonance imaging, Software, Computer science

## Abstract

There is little evidence on the applicability of deep learning (DL) in the segmentation of acute ischemic lesions on diffusion-weighted imaging (DWI) between magnetic resonance imaging (MRI) scanners of different manufacturers. We retrospectively included DWI data of patients with acute ischemic lesions from six centers. Dataset A (n = 2986) and B (n = 3951) included data from Siemens and GE MRI scanners, respectively. The datasets were split into the training (80%), validation (10%), and internal test (10%) sets, and six neuroradiologists created ground-truth masks. Models A and B were the proposed neural networks trained on datasets A and B. The models subsequently fine-tuned across the datasets using their validation data. Another radiologist performed the segmentation on the test sets for comparisons. The median Dice scores of models A and B were 0.858 and 0.857 for the internal tests, which were non-inferior to the radiologist’s performance, but demonstrated lower performance than the radiologist on the external tests. Fine-tuned models A and B achieved median Dice scores of 0.832 and 0.846, which were non-inferior to the radiologist's performance on the external tests. The present work shows that the inter-vendor operability of deep learning for the segmentation of ischemic lesions on DWI might be enhanced via transfer learning; thereby, their clinical applicability and generalizability could be improved.

## Introduction

Ischemic stroke is a significant public health problem and one of the leading causes of mortality and disability worldwide^1^. Ischemic stroke is routinely diagnosed using neuroimaging modalities, such as computed tomography (CT) and magnetic resonance imaging (MRI)^1^. Given that CT is widely available and has a shorter acquisition time, it is now recommended to use CT over MRI due to the importance of initiating treatment early^2^. Nevertheless, MRI offers valuable information in challenging cases and better delineation of ischemic lesions in the early stages of the disease due to its unsurpassed contrast resolution^3,4^. Furthermore, the volume of the ischemic core assessed using diffusion-weighted imaging (DWI) provides essential insights for decision-making. DWI is beneficial by allowing evaluation of the vascular territory of the stroke lesions, predicting whether a patient with stroke will be benefited from the treatment^5–7^. Also, it might serve as a potential non-invasive biomarker for predicting stroke-related long-term complications^8^. The current gold standard for measuring the ischemic core on DWI is manual segmentation, which is a labor-intensive, time-consuming, and tedious task; therefore, it may be omitted in daily practice due to the need for instantaneous estimation. Accordingly, several traditional machine learning- and threshold-based approaches have been proposed to segment acute ischemic lesions with variable success rates^9,10^.

Deep learning (DL) is a subfield of machine learning that involves using a stack of interconnected neurons to simultaneously extract the representative features and make predictions for a given task^11^. Recent studies have demonstrated that DL, particularly convolutional neural networks (CNNs), is a robust tool for analyzing medical images for various tasks, including classification, segmentation, and object detection^12^. Several studies published within the last three years have demonstrated the yield of deep learning in estimating the ischemic core on DWI and that DL-based methods surpassed the predecessor methods mentioned above^13–19^. However, most of the earlier efforts that utilized DL used samples obtained at a single institution and lacked independent external validation and performance comparison with a radiologist^13–17^. Despite the high-representative capacity of DL models, several authors have recently criticized the generalizability of DL models across datasets derived from different domains (e.g., MRI obtained with different MRI scanners or at different institutions)^20,21^.

In this study, we used a novel DL architecture, residual two-dimensional (2D) U-net with convolutional long short-term memory (ConvLSTM) unit, for automated segmentation of acute ischemic lesions on DWI using a large-scale multicenter dataset. The aims of this study were the following: first, to externally validate the performance of DL models across different MRI vendors; second, to compare the diagnostic performance of the DL models with that of a radiologist; and third, to investigate the benefits of transfer learning that involves evaluating whether a small amount of data derived from the target domain would improve the performance of DL models across different MRI vendors.

## Materials and methods

**Figure 1:**
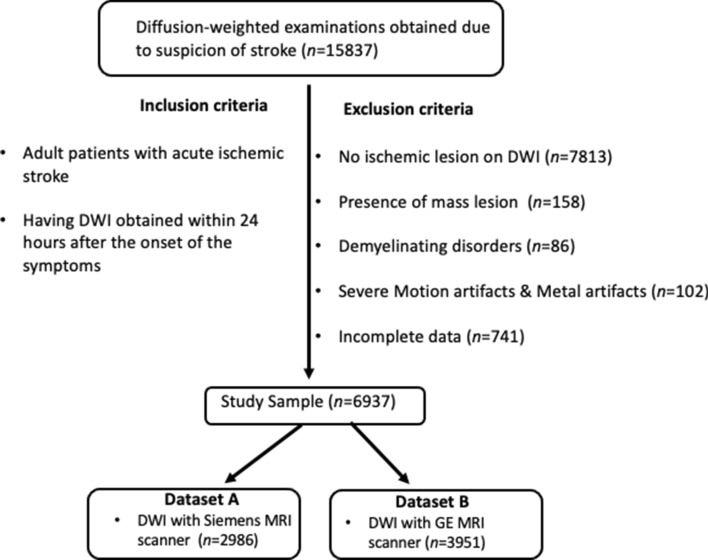
The patient selection process in the current study.

Istanbul Mehmet Akif Ersoy Research and Training Hospital Ethics committee approved this retrospective multicenter study (Approval number: 2019-77) and waived the need for informed consent. All procedures performed in studies involving human participants were in accordance with the ethical standards of the institutional and/or national research committee and with the 1964 Helsinki declaration and its later amendments or comparable ethical standards. The dataset was obtained from six tertiary care centers. Six medical doctors reviewed the radiology reports of consecutive brain DWIs of adult patients obtained with clinical suspicion of acute ischemic stroke between January 2012 and October 2019 using several keywords (e.g., stroke, ischemia, limb weakness, and diffusion-restriction). The exclusion criteria were as follows: (1) DWI obtained 24 h after the onset of the symptoms; (2) patients with a primary brain tumor, metastatic brain tumors, or demyelinating lesions; (3) DWI with severe motion or metallic artifacts; and (4) incomplete imaging or clinical data (i.e., no information on the ischemia time or lack of high b-value images or apparent diffusion coefficient maps). Detailed information regarding the patient selection process is depicted in Fig. 1.

Of the six study centers, three had 1.5 T Genera Electronics MRI scanners (Optima MR450w, Signa HDxt, and Signa Explorer; GE Healthcare, Milwaukee, WI, USA) and the other three had 1.5 T Siemens scanners (Aera, Avanto, and Symphony, Siemens Healthineers, Erlangen, Germany). The data of the six centers were divided into two parts based on the MR scanner; dataset A included DWIs obtained using a Siemens scanner, and dataset B included DWIs obtained with a GE scanner. Detailed information regarding the DWI protocols at each center is provided in Supplementary Table S1.

### Ground-truth segmentations

Six neuroradiologists (E.K., O.K., H.H.S., B.K., S.N.D., M.V.) with over ten years of neuroradiology experience from each center examined the recruited images. The neuroradiologists were free to assess all the available clinical and radiological data during the evaluation. Briefly, the neuroradiologist evaluated the images for acute ischemia; acute ischemic lesions were defined as those with a hyperintense signal on high b-value diffusion-weighted (DW) images and corresponding hypointensities on apparent diffusion coefficient (ADC) maps^22^. If a neuroradiologist decided that a scan had undiagnostic image quality (i.e., severe motion or metallic artifacts) or had no visible acute ischemic lesion, then the patient was excluded from the study.

Subsequently, DW images with the highest b-values and corresponding ADC maps of the patients were anonymized. The formula of the ADC calculation map was as follows: ADC =  − ln (S/S0)/b, where S refers to the signal intensity of the higher b-value image, S0 is the signal intensity of image with no diffusion gradients (i.e., b0) and b is the b value. A unique identification number was assigned to each patient for further analyses. Anonymized ADC maps and DWIs were imported into a known open-source software for segmentation (ImageJ, https://imagej.nih.gov).

The neuroradiologists performed segmentations on the DW images using a free-hand region of interest. The segmentation quality of the test sets of datasets A and B was mandatory to achieve reliable performance comparisons. The neuroradiologists re-drew the segmentations on the same images of the test sets in two different sessions after an interval of 1 month. To this end, each patient in the test sample had three different segmentation masks provided by the same neuroradiologists. An intra-reader majority voting was used to create ground-truth masks of the test sets. The pixels accounted as positive for an ischemic lesion in two or more masks were accepted as positive, and those accounted as negative for an ischemic lesion in two or more masks were accepted as negative.

### DL models

We employed a well-known CNN architecture for biomedical image segmentation, U-net, or U-shaped networks, but made several modifications^23^. The original U-net model has two main components: the encoder, which serves to identify the most representative features of the images, and the decoder, in which the up-sampling process is performed to regain spatial resolution while preserving the high-representative power of the feature maps for precise segmentation. The concatenations between the encoder and decoder facilitate the network’s ability to preserve the spatial information of the pixels. U-net can work on both 2D and three-dimensional (3D) data^23,24^.

**Figure 2:**
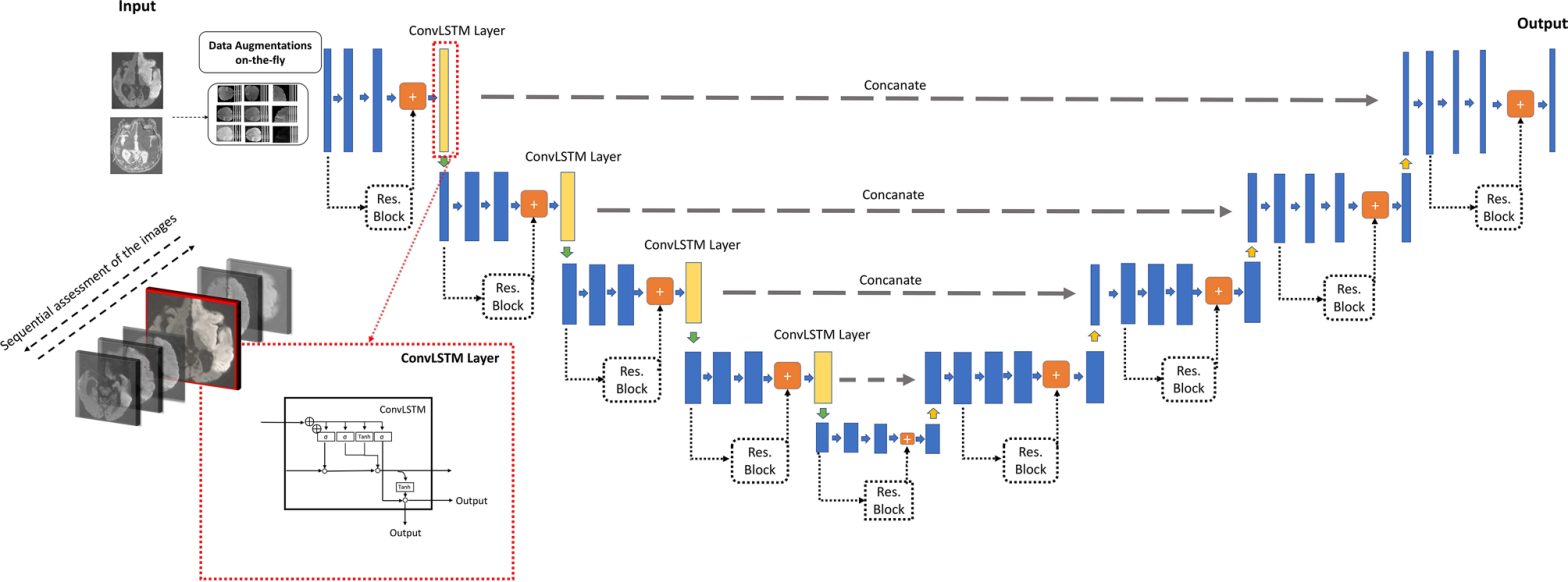
Residual two-dimensional convolutional long short-term memory (ConvLSTM) U-net. The stack of high b-value diffusion-weighted images and corresponding apparent diffusion coefficient maps are fed into the network per-patient. Two important modifications are made to the 2D U-net. First, residual layers are utilized for each convolutional block, which allows unimpeded propagation of information throughout the network and mitigates the vanishing gradient problem. Second, bi-directional ConvLSTM layers are implemented on top of each convolutional block of the encoder network to allow communication of the feature maps. Consequently, it enables the network to consider all of the slices of an examination before delineating an ischemic lesion’s borders. Therefore, we suggest that this architecture, to some extent, mimics how radiologists assess images, which involves sequential assessment of all slices of an examination before making the final diagnosis or, in this context, performing segmentation.

In the present work, we used a residual ConvLSTM U-Net, which is a hybrid network architecture that leverages the high spatial and sequential representational capacity of convolutional and recurrent neural networks as well as exploits the skip connections that facilitate information flow throughout the network^25,26^. Figure 2 illustrates the details of the residual ConvLSTM U-Net architecture.

### DL experiments

The stack of high b-value DW images and corresponding ADC maps were fed into the network on a patient basis using two different channels. Following typical image pre-processing operations were performed on the images before feeding them into the network: (1) intensity normalization within 0–1; (2) resampling the images into 224*224 pixels with a voxel resolution of 1.375*1.375*4 mm^3^; (3) and image windowing, which is determined as the best window level for the neuroradiologist's eye for assessing DWIs for each center and scanner. Several data augmentations, rotation, flipping, and elastic deformations were implemented on the go.

All DL experiments were conducted using a high-level DL library, Keras on TensorFlow (Tensorflow 1.4 Google LLC, Mountain View, CA). The total trainable parameters of the residual ConvLSTM U-Net were 8,228,401. The hyperparameters of the models were optimized using the validation partition and were as follows: loss function was Tversky loss (alpha = 0.7, Beta = 0.3); the number of epochs was 100; optimizer was Adam; learning rate was 1e-5; the batch size was 2. The total training time for models A and B was 10.5 and 12 h, respectively.

Datasets A and B were split into three parts as the training (80%), validation (10%), and internal test (10%) sets. The best model was selected based on its performance on the validation data. The DL models trained on datasets A and B were referred to as models A and B. The segmentation performance of models A and B was first assessed on the internal test sets consisting of images from the same manufacturer. Subsequently, their performances were evaluated on the test partition of the other dataset (i.e., model A on the test set of dataset B and vice versa), and these assessments were referred to as external tests.

**Figure 3:**
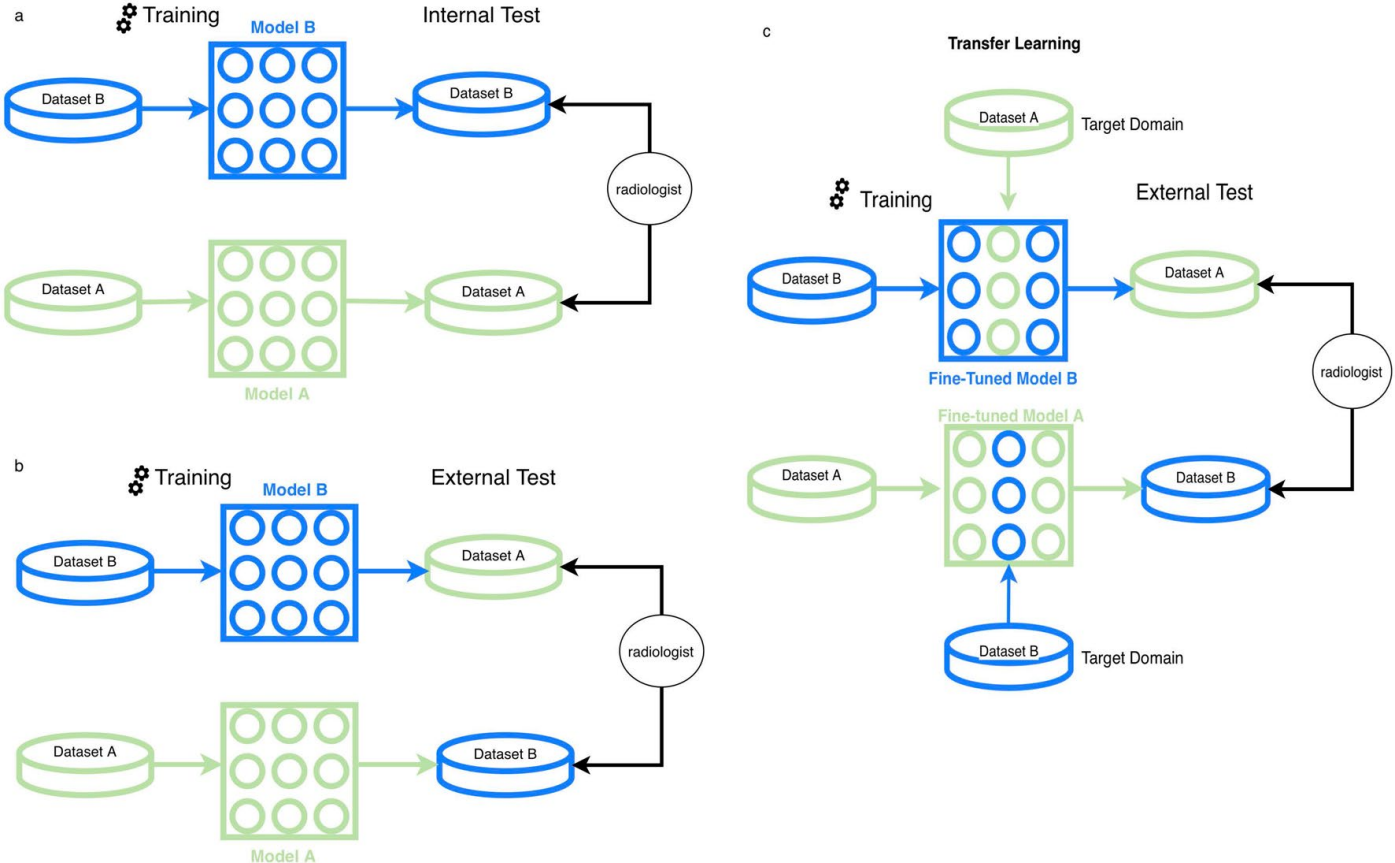
The flowchart of the deep learning experiments. **(A)** The deep learning models trained on datasets A and B were referred as models A and B. The segmentation performance of models A and B were first assessed on the internal test sets that consisted of images from the scanners of the same manufacturer. **(B)** Subsequently, the models’ performances were evaluated on the test partition of the other dataset, which was referred as external test. **(C)** The validation parts of each dataset were used to fine-tune the pre-trained model using transfer learning. These models were referred as fine-tuned models A and B, respectively. Subsequently, the fine-tuned models’ performances were assessed on the external test sets. A single expert radiologist made segmentations on the test partitions of the datasets for performance comparisons with the deep learning models.

Furthermore, to simulate a scenario of extensive available imaging data from one manufacturer while it is limited from another, we utilized transfer learning^27^. The validation parts of each dataset were used to fine-tune the pre-trained model on the other dataset (e.g., pre-trained model A was fine-tuned with the validation part of dataset B and vice versa) for approximately 20 epochs with a learning rate of 1e-6. These models were referred to as fine-tuned models A and B, respectively. Figure 3 shows the DL experiment pipeline of the present work.

### Evaluating the performances

The primary metric for investigating a model's performance was the Dice coefficient (two * areas of overlap/total pixels combined), which is a measure of overlap between the model’s predictions and the ground truth^28^. The Dice coefficient ranges between 0 and 1, where 1 represents a complete match between the ground truth and prediction, while 0 reflects no match. To compare the performance of the DL models with that of a radiologist, another radiologist with eight years of experience (D.A.) manually delineated the borders of the ischemic lesions on DWI on the test partitions.

### Statistical analysis

Statistical analysis was performed using Scipy library v1.5.4 of Python programming language (“https://docs.scipy.org”). Categorical variables are presented as frequencies and percentages. Continuous variables were investigated using distribution plots and the Shapiro–Wilk test to assess for normality. Normally distributed continuous variables are presented as mean, standard deviations, and ranges, while non-normally distributed continuous variables are presented as median and interquartile ranges. Mann–Whitney U test was used to compare each model’s performance on its internal and external tests. Wilcoxon test was used to compare the performances of fine-tuned and native models A and B on the external test sets. Mann–Whitney U test was used to compare the Dice scores of the models and the radiologist on the test sets. A Spearman's rank-order correlation was run to assess the correlations between the manually measured and predicted ischemic lesion volumes. The Spearman r values were interpreted as follows: r of < 0.30 as a negligible correlation, 0.30–0.50 as a low correlation, 0.50–0.70 as a moderate correlation, 0.70–0.90 as a high correlation, and > 0.90 as a very high correlation^29^. A p-value < 5% was considered as a statistically significant result.

### Ethical statement and consent to participate

All procedures performed in studies involving human participants were in accordance with the ethical standards of the institutional and/or national research committee and with the 1964 Helsinki declaration and its later amendments or comparable ethical standards.

## Results

Dataset A consisted of 2986 patients (1793/2986 men, 60%) with a mean age of 65.80 ± 11.28 years (range, 47–85), and dataset B comprised 3951 patients (2223/3951 men, 56.26%) with a mean age of 63.07 ± 10.78 years (range, 45–81). The training, validation, and internal test samples of datasets A and B included 2385, 301, and 300 images and 3157, 397, and 397 images, respectively. The median volumes of ischemic lesions were 3.95 mL (2.61–17.44), 3.41 mL (2.43–15.04), and 2.64 mL (2.19–4.68) for the training, validation, and test sets of dataset A. The median volumes of ischemic lesions were 3.27 mL (2.63–6.94), 3.21 mL (2.58–6.25), 3.18 mL (2.55–6.42) for the training, validation, and test sets of dataset B. The median time interval between the onset of symptoms and imaging was 6.5 h (interquartile range, IQR, 4.5–8) and 7 h (IQR, 6–8) in datasets A and B, respectively. Detailed information on the study sample is provided in Table 1.

**Table 1:** The detailed characteristics of the study sample.

Variables	Dataset A (n = 2986)	Dataset B (n = 3951)
**Age (years)**		
Total dataset	65.80 ± 11.28 (47–85)	63.07 ± 10.78 (45–81)
Training set	65.81 ± 11.23 (47–85)	63.01 ± 10.78 (47–85)
Validation set	67.09 ± 11.29 (48–84)	63.83 ± 10.98 (45–72)
Test set	65.26 ± 11.64 (51–81)	62.76 ± 10.52 (45–71)
**Male gender (%)**		
Total dataset	1793/2986 (60%)	2223/3951 (56.26%)
Training set	1484/2385 (62.22%)	1833/3157 (58.06%)
Validation set	159/301 (52.82%)	204/397 (51.38%)
Test set	150/300 (50%)	197/397 (49.62%)
**Symptoms to MRI (hours)**		
Total dataset	6.5 (4.5–8)	7 (6–9)
Training set	6 (4.5–8)	7 (6–9)
Validation set	5.5 (4.5–7.5)	6.5 (5–10)
Test set	7.5 (6–10)	8 (3–11)
**Manual lesion volumes (mL)**		
Total dataset	3.66 (2.51–15.55)	3.25 ( 2.62–6.81)
Training set	3.95 ( 2.61–17.44)	3.27 (2.63–6.94)
Validation set	3.41 ( 2.43–15.04)	3.21 ( 2.58–6.25)
Test set	2.64 (2.19–4.68)	3.18 (2.55–6.42)

Continuous variables are presented with the mean ±  standard deviations or median and interquartile ranges.

The median Dice scores of model A were 0.866 (IQR, 0.774–0.918), 0.835 (IQR, 0.702–0.899), and 0.858 (IQR, 0.752–0.909) in the training, validation, and internal test tests, respectively. The model A predicted lesions volumes 4.92 mL (2.76–18.4), 4.71 mL (2.55–15.52), and 3.24 mL (2.09–6.20) for the respective parts. The Spearman correlation test revealed high positive correlations between manually measured and predicted volumes for the training, validation, and internal test sets (r = 0.89, r = 0.84, r = 0.73, P < 0.0001 for all).

The median Dice scores of model B were 0.896 (IQR, 0.813–0.940), 0.865 (IQR, 0.745–0.923), and 0.857 (IQR, 0.723–0.921) in the training, validation, and internal test tests, respectively. The model B predicted lesions volumes as 3.86 mL (2.73–8.52), 3.74 mL (2.72–7.89), and 3.93 mL (2.68–8.79) for the respective parts. The Spearman correlation test revealed moderate to high positive correlations between manually measured and predicted volumes for the training, validation, and internal test sets (r = 0.77, r = 0.67, r = 0.68, and P < 0.0001 for all).

Model A yielded a median Dice score of 0.734 (IQR, 0.56–0.843) in the external test consisting of images from dataset B. There was a significant difference between model A’s performance on the internal and external test sets in Dice scores (0.866 vs. 0.734, respectively, P < 0.0001). Model A predicted lesion volumes as 2.15 mL (1.48–4.15) in the external test set, and the Spearman correlation test revealed a moderate positive correlation between manually measured and predicted volumes (r = 0.58, P < 0.0001). Further detailed metrics regarding model B performance are given in Table 2.

**Table 2:** Diagnostic performances of Models A and B.

	Dice score	Recall	Precision
**Model A**			
Training	0.866 (0.774–0.918)	0.953 (0.851–0.992)	0.736 (0.658–0.780)
Validation	0.835 (0.702–0.899),	0.918 (0.772–0.967)	0.710 (0.596–0.764)
Internal test	0.858 (0.752–0.909)	0.944 (0.828–0.981)	0.639 (0.729–0.773)
External test without TF	0.734 (0.56–0.843)	0.807 (0.616–0.927)	0.624 (0.476–0.716)
External test with TF	0.832 (0.671–0.916)	0.915 (0.738–0.978)	0.707 (0.57–0.779)
**Model B**			
Training	0.896 (0.813–0.940),	0.970 (0.887–0.992)	0.771 (0.7–0.808)
Validation	0.865 (0.745–0.923)	0.943 (0.813–0.983)	0.744 (0.641–0.794)
Internal test	0.857 (0.723–0.921)	0.934 (0.787–0.970)	0.737 (0.622–0.792)
External test without TF	0.756 (0.613–0.851)	0.824 (0.668–0.928)	0.650 (0.527–0.732)
External test with TF	0.846 (0.730–0.902)	0.922 (0.788–0.969)	0.727 (0.638–0.776)

Continuous variables are presented with median and interquartile ranges.

*TF* transfer learning.

Model B yielded a median Dice score of 0.756 (IQR, 0.613–0.851) in the external test consisting of images from dataset A. There was a significant difference between model B’s performance on the internal and external test sets in Dice scores (0.857 vs. 0.756, respectively, P < 0.0001). Model B predicted lesion volumes as 3.57 mL (2.62–6.09) in the external test set, and the Spearman correlation test revealed a moderate positive correlation between manually measured and predicted volumes (r = 0.60, P < 0.0001). Further detailed metrics regarding model B performance are given in Table 2.

### Radiologist versus DL

The radiologist achieved a median Dice score of 0.868 (IQR, 0.762–0.895) and 0.867 (IQR, 0.733–0.899) on the test partitions of datasets A and B, respectively. Model A’s performance, compared with that of the radiologist, on dataset A’s internal test was not significantly different (0. 868 vs. 0.858, respectively, P = 0.273). Similarly, model B’s performance, compared with that of the radiologist, on dataset B’s internal test was not significantly different (0. 867 vs. 0. 857, respectively, P = 0.668). Furthermore, the models’ performances on the external test sets were compared with those of the radiologist. The Mann–Whitney U model identified that models A and B demonstrated significantly lower performance than that the radiologist on the external tests. Model A yielded a median Dice score of 0.734 (IQR, 0.56–0.843), while the radiologist achieved a median Dice score of 0.867 (IQR, 0.733–0.899) on the test partition of dataset B (P < 0.0001). Model B yielded a median Dice score of 0.756 (IQR, 0.613–0.851), while the radiologist achieved a median Dice score of 0.868 (IQR, 0.762–0.895) on dataset A’s test partition (P < 0.0001).

### DL with transfer learning

Fine-tuned model A achieved a median Dice score of 0.832 (IQR, 0.671–0.916) when applied to the external test consisting of images from dataset B. Wilcoxon test revealed that fine-tuned model A achieved statistically higher performance than native model A on the external test (0.818 vs. 0.734, respectively, P < 0.0001). In addition, fine-tuned model A demonstrated non-inferior performance compared with that of the radiologist (0.832 vs. 0.867, respectively, P = 0.127). Fine-tuned model A predicted lesion volumes as 2.45 mL (1.78–4.45) in the external test set, and the Spearman correlation test revealed a moderate positive correlation between manually measured and predicted volumes (r = 0.65, P < 0.0001).

**Figure 4:**
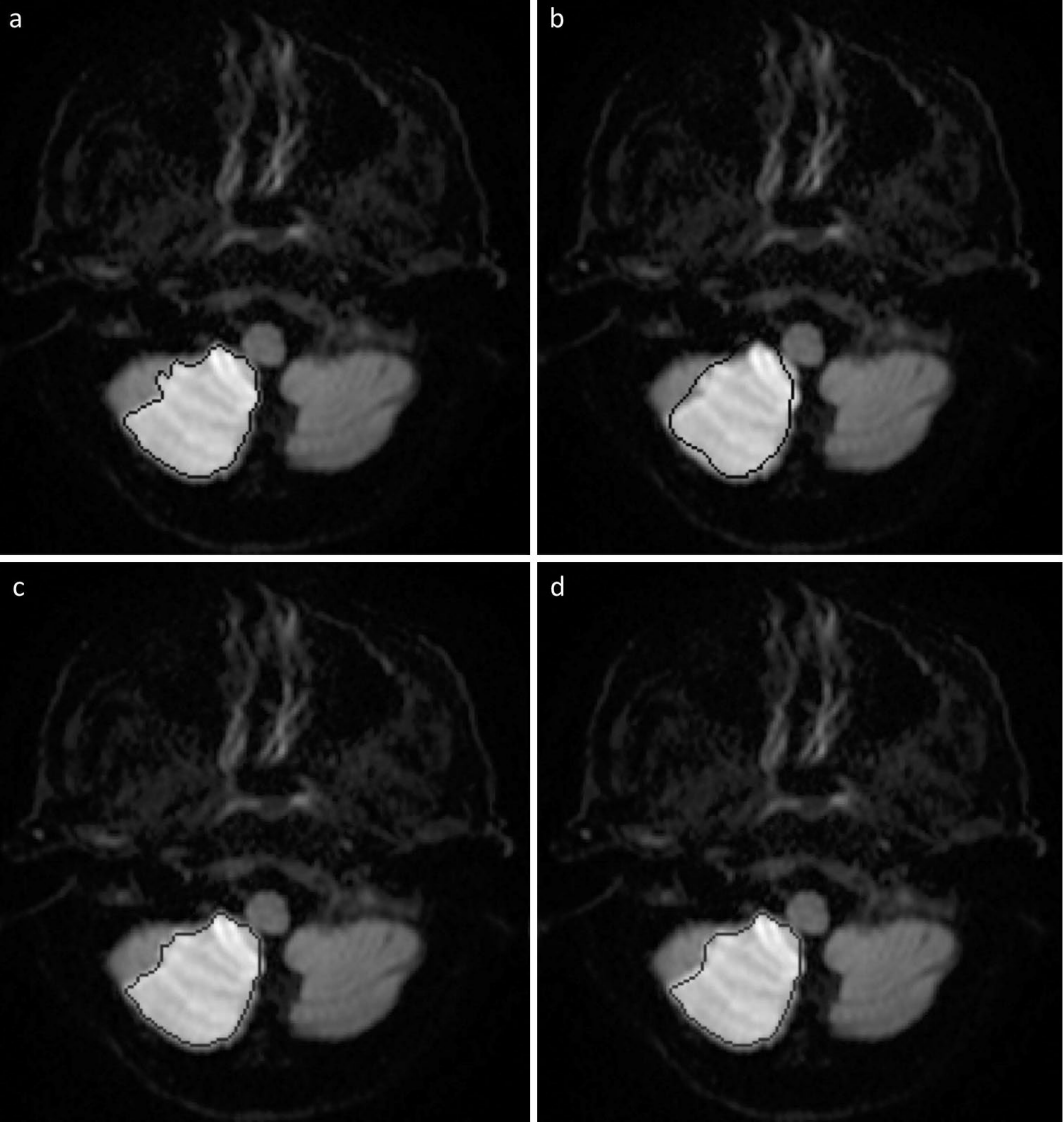
Acute ischemic lesion segmentation of model B on the external test. Figures shows images of a 64-year-old male patient with acute ischemic lesion in the right cerebellar hemisphere. **(A)** Ground-truth segmentation mask created by the neuroradiologist. **(B)** The segmentation of native model B. Note the incorrect contours, which is more profoundly marked in the lateral part of the lesion. **(C)** The segmentation by the radiologist. **(D)** The segmentation by fine-tuned model B. Note that the fine-tune model demonstrates similar segmentation performance to that of the radiologist, while delineating the borders of the lesion much correctly than the native model.

Fine-tuned model B achieved a median Dice score of 0.846 (IQR, 0.730–0.902) when applied to the external test consisting of images from dataset A. Fine-tuned model B yielded a higher performance compared with native model B on the external test (0.846 vs. 0.756, respectively, P < 0.0001). In addition, fine-tuned model B demonstrated non-inferior performance compared with that of the neuroradiologist (0.846 vs. 0.868, respectively, P = 0.468). Fine-tuned model B predicted lesion volumes as 3.45 mL (2.5–5.97) in the external test set, and the Spearman correlation test revealed a high positive correlation between manually measured and predicted volumes (r = 0.71, P < 0.0001). Figure 4 illustrates the segmentation of the DL model.

## Discussions

In the present work, a residual ConvLSTM U-Net was applied for the segmentation of acute ischemic lesions on DWI using a large-scale multicenter dataset to investigate the performance of the DL models across different MRI manufacturers. This study makes several contributions to the existing literature on DL-based acute ischemic lesion segmentation on DWI. (1) The DL models demonstrated non-inferior performance compared with a radiologist in delineating borders of ischemic lesions when applied to previously unseen images derived from the same manufacturer on which the models were previously trained. (2) The DL models yielded reasonable performance when applied to DW images derived from a different MRI manufacturer with a median Dice score of 0.734–0.756. (3) However, the segmentation performance of the DL models was substantially higher when applied to previously unseen DW images of the same manufacturer compared with that of DL models applied to previously unseen images of a different manufacturer. (4) In contrast, the radiologist’s performance did not differ between datasets derived from different MRI manufacturers. (5) When the DL models were fine-tuned with a small fraction of the images obtained from the other manufacturer using transfer learning, their performance increased substantially to a non-inferior level to that of a radiologist on the external tests. (6) DL model-based infarct volume estimations yielded moderate to high positive correlations with the ground-truth volume measurements. Likewise, transfer learning improves DL models’ performances in measuring infarct volumes.

To the best of our knowledge, the present work had the largest sample size reported thus far. The systematic evaluation of the contribution of transfer learning for domain adaptation across multicenter expert annotated datasets was the prominent uniqueness of the present work. Comparing DL's segmentation performance with radiologists across MRI scanner vendors was the other unique contribution to the prior literature. Besides, the present work was the first to use ConvLSTM block on DWI and ADC maps in stroke imaging, as far as we know. The standard convolutional block could not aggregate the information on the z-axis of the radiological images, which often is mandatory to make the diagnosis correctly^30,31^. The ConvLSTM blocks allow assessing DWI and ADC slices with an arbitrary range while capturing inter-slice dependencies^26^.

We do acknowledge that ConvLSTM hypothetically might be inferior compared with the 3D variants of U-Net in assessing cross-sectional medical data. Some prior works suggest that 3D variants of U-Nets have outstanding performance in many medical image segmentation tasks^30^. However, it has been shown that 3D models showed their best performance when the voxels are isotropic (i.e., the same voxel size across three dimensions), yet their performance might significantly drop on the images with anisotropic voxel sizes^31^. Brain DWI often are highly anisotropic images since the slice thickness (i.e., z-axis) generally is much more than the in-plane spatial resolution (i.e., y and x-axes). Additionally, 3D U-net requires higher memory capacity, and it might require lowering the original spatial resolution or using patch-based approaches in the network, which inevitably leads to loss of contextual information^32,33^. Nevertheless, head-to-head comparisons of 3D U-net and ConvLSTM U-net in segmenting stroke lesions on DWI should be systematically explored in future work.

Chen et al.^19^ were some of the first to demonstrate the potential of DL algorithms in ischemic lesion segmentation on DWI on a large scale. The authors proposed two sequential CNNs, an ensemble of DeconvNets followed by a multi-scale CNN. The authors reported a mean Dice coefficient of 0.67 in 741 participants. However, all their images were derived from an MRI scanner of the same manufacturer. Kim et al.^16^ used a 2D U-net to segment acute ischemic lesions on DWI with corresponding ADC maps in 296 participants and compared its performance with a commercially available stroke imaging software. The authors demonstrated that their DL model achieved similar performance to that of the commercially available software. However, the study was limited by the small sample size and the domination of DWIs from a single vendor. Zhang et al.^14^ investigated the performance of deep 3D fully convolutional DenseNets for ischemic lesion segmentation on DWI and ADC in 242 patients. The authors were obligated to resample the original spatial resolution of the images to a lower dimension to use a deep 3D model due to memory constraints but achieved excellent performance with a mean Dice similarity coefficient of 0.79. Their dataset was derived from MRI scanners of two vendors; however, the authors did not mention the distribution of the images regarding the manufacturer.

In a large-scale multicenter international study by Wu et al.^18^, an ensemble 3D CNN, DeepMedic framework, was used to segment acute ischemic lesions on DWI with their ADC maps in a heterogeneous cohort. Our study shared similar foundations with their work because they also tried to demonstrate the applicability of DL in a diverse patient population to reveal its usefulness in practice. The authors included 2770 patients, but the ground-truth segmentations were only available for approximately a quarter of the data. The authors achieved a mean Dice score of 0.77 and highlighted the benefits of using the ensemble. Furthermore, they provided essential insights into the use of DL for acute ischemic lesion segmentation on DWI, such as the negative effects of different b-values on a model's segmentation performance and the robustness of DL models across different field strengths.

We acknowledge that the heterogeneous nature of the acute ischemic lesions, in addition to the technical variations and differences in the DL architectures used to evaluate the lesions, inevitably hampers the comparability of studies on DL-based ischemic lesion segmentation. In recent years, there have been some attempts, such as the ischemic lesion segmentation challenge, to create publicly available datasets for investigating the performance of DL models^34^. However, the existing datasets were of small sizes and lacked variability regarding MRI scanner vendors^34^. Therefore, we suggest that it is impossible to compare the performance of the proposed DL-based solutions for acute ischemic lesion segmentation.

Nevertheless, we did not aim to compare the performances of our DL models with those proposed in the earlier works but rather aimed to assess the inter-vendor operability of these models. This is of clinical importance since it is not an unlikely clinical or academic scenario in which a trained DL model should be directly used at a center in which the MRI scans are routinely obtained using a scanner from a different manufacturer. As expected, a radiologist did not demonstrate substantial impairment in terms of segmentation performance across different vendors. In contrast, the performance of the DL models was impaired when applied to DW images obtained using a different vendor’s scanner.

To this end, we used transfer learning to improve the model’s performance across different MR vendors. The DL models that were fine-tuned using only a small part of the dataset from different vendors demonstrated substantial performance improvements. In addition fine-tuned models demonstrated non-inferior performance in acute ischemic lesion segmentation compared with that of an expert radiologist. These findings imply that a base DL model could be readily available across different MRI scanners of manufacturers if it is provided with a relatively small amount of data from the target domain. However, we admit that transfer learning might be beneficial only to some degree in such scenarios because it also requires some labeled images from the target domain. To this end, several unsupervised solutions have been proposed in the context of domain adaptation and generalization, which remains an active research area^35^.

Nevertheless, several limitations to this study should be acknowledged. Our dataset consisted of only two MRI vendors; therefore, the inter-operability of the DL models across other vendors could not be evaluated. Second, the exclusion criteria (e.g., DW images with severe motion artifacts or tumors) of the present work might result in a bias toward the DL models such that an experienced radiologist might not misdiagnose and delineate the borders of a brain tumor on DW. In contrast, the DL model might not be as accurate as of the expert since it was trained for a narrower task. Therefore, DL models might provide inferior performance to an experienced radiologist even when applied to the same MR vendor's images. In the same vein, we did not investigate DL models’ performance on normally appearing DW images; hence, potentially false-positive predictions on such cases might also turn the comparison in favor of radiologists. Third, the present work dataset did not include any DW images obtained with a 3 T MR scanner; therefore, we could not compare the applicability of these models across different field strengths. Fourth, we feed the network with only using the DW images with the highest b-values, yet simultaneous use of the DW images with lower b-values or b0 images might improve the segmentation performance. We suggest that future studies should investigate the combination of DW images with different b-values while feeding the DL models to reveal whether it gives any segmentation performance boosts.

In conclusion, the DL model, residual ConvLSTM U-Net, demonstrated non-inferior performance to an expert radiologist in segmenting acute ischemic lesions on DWI when applied to previously unseen images derived from the same manufacturer on which the models were previously trained, but its yields worsened across different manufacturers. Notably, fine-tuning the model using a small sample of the images from the different manufacturer (i.e., target domain) substantially increased its performance to a non-inferior level compared with an expert radiologist.

## Supplementary Information

Supplementary Table S1.

## Data Availability

Fine-tuned models and several examples of test images are provided in the http://52.29.179.238:8501/. The researchers could upload their anonymized high b-value diffusion-weighted images as zip files to the website to test models’ performance on their own images. Further data access requests by qualified researchers trained in human subject confidentiality protocols should be sent to the corresponding author.
