# Supplementary material for: Inter-vendor performance of deep learning in segmenting acute ischemic lesions on diffusion-weighted imaging: a multicenter study
Source: Sci Rep. 2021 Jun 14;11:12434. doi: 10.1038/s41598-021-91467-x (PMC8203621; doi:10.1038/s41598-021-91467-x)
Supplement: Supplementary file 1 — Supplementary Table S1. [file 41598_2021_91467_MOESM1_ESM.docx]

**Inter-vendor performance of deep learning in segmenting acute ischemic lesions on diffusion-weighted imaging: a multicenter study**

Deniz Alis^1^, Mert Yergin^2^, Ceren Alis^3^, Cagdas Topel^4^, Ozan Asmakutlu^4^, Omer Bagcilar^5^, Yeseren Deniz Senli^6^, Ahmet Ustundag^6^, Vefa Salt^6^, Sebahat Nacar Dogan^7^, Murat Velioglu^8^, Hakan Hatem Selcuk^9^, Batuhan Kara^9^, Ilkay Oksuz^10^, Osman Kizilkilic^6^, Ercan Karaarslan^1^

Deniz Alis, Assistant Professor, [drdenizalis@gmail.com](mailto:drdenizalis@gmail.com) (Corresponding Authors)

Mert Yergin, mertmyergin@gmail.com

Ceren Alis, M.D., cerencivcik@gmail.com

Cagdas Topel, M.D., cgdstpl@gmail.com

Ozan Asmakutlu, M.D., ozanasmakutlu@hotmail.com

Omer Bagcilar, M.D., omerbagcilar@hotmail.com

Yeseren Deniz Senli, M.D., yeserendsenli@hotmail.com

Ahmet Ustundag, M.D., ahmet_ustundag78@hotmail.com

Vefa Salt, M.D., vefasalt@gmail.com

Sebahat Nacar Dogan, M.D., sebahatdogan@yahoo.com

Murat Velioglu, Associate Professor, velix@hotmail.com

Hakan Hatem Selcuk, Associate Professor, hatemhakanselcuk@gmail.com

Batuhan Kara, Associate Professor, [kara_batuhan@yahoo.com](mailto:kara_batuhan@yahoo.com)

Ilkay Oksuz, Assistant Professor, oksuzilkay@gmail.com

Osman Kizilkilic, Professor, osmank@istanbul.edu.tr

Ercan Karaarslan, Professor, ercankaraarslan@yahoo.com

*^1^*Acibadem Mehmet Ali Aydinlar University School of Medicine, Radiology Department, Istanbul/Turkey

^2^Bahcesehir University, Department of Software Engineering and applied sciences, Istanbul/Turkey

^3^ Istanbul University-Cerrahpasa, Cerrahpaşa Medical Faculty, Neurology Department, Istanbul/Turkey

^4^ Istanbul Mehmet Akif Ersoy Thoracic and Cardiovascular Surgery Training and Research Hospital, Department of Radiology, Halkali/Istanbul, TURKEY

^5^ Istanbul Silivri State Hospital, Radiology Department, Istanbul/Turkey

^6^ Istanbul University-Cerrahpasa, Cerrahpaşa Medical Faculty, Radiology Department, Istanbul/Turkey

^7^ Istanbul Gaziosmanpasa Training and Research Hospital, Radiology Department, Istanbul-Turkey

^8^ Istanbul Fatih Sultan Mehmet Training and Research Hospital, Radiology Department, Istanbul-Turkey

^9^ Istanbul Bakırköy Sadi Konuk Training and Research Hospital, Radiology Department, Istanbul-Turkey

^10^Istanbul Technical University, Department of Software Engineering and applied sciences, Istanbul/Turkey

**Corresponding Author: Deniz Alis, M.D. Acibadem Mehmet Ali Aydinlar University, Department of Radiology, Atasehir/ Istanbul, TURKEY Phone number: +90 5364797429 drdenizalis@gmail.com**

**Short Title:** Deep learning in MRIs of different manufacturers

**Type of article**: Original research

**Total number of figures/tables: Figures: 4/ Table: 1**

**Total word count: 3547 (main text)**

**Table S1**. DWI protocols of the study.

|  | Center 1 | | Center 2 | Center 3 | Center 4 | | Center 5 | | Center 6 |
| --- | --- | --- | --- | --- | --- | --- | --- | --- | --- |
| Parameters | Siemens Avanto | Siemens Symphony | Siemens  Aera | Signa Explorer | Optima MR450w | Signa HDxt | Optima MR450w | Signa Explorer | Siemens Avanto |
| Axial | Axial | Axial | Axial | Axial | Axial | Axial | Axial | Axial | Axial |
| Field strength | 1.5 | 1.5 | 1.5 | 1.5 | 1.5 | 1.5 | 1.5 | 1.5 | 1.5 |
| Head coil channels | 12 | 8 | 16 | 12 | 12 | 12 | 12 | 12 | 16 |
| TR (ms) | 3200 | 3500 | 5500 | 7100 | 5000 | 6500 | 5000 | 5000 | 3500 |
| TE (ms) | 80 | 110 | 125 | 105 | 85 | 95 | 90 | 85 | 90 |
| b-values | 0,500,1000 | 0,500,1000 | 0,1500 | 0,1000 | 0,1000 | 0,1000 | 0,1000 | 0,1000 | 0,500,1000 |
| FoV (mm) | 230x230 | 230x230 | 230x230 | 220x220 | 220x220 | 220x220 | 220x220 | 220x220 | 230x230 |
| Voxel size (mm) | 1.8x1.8x5 | 1.8x1.8x5 | 1.8x1.8x5 | 1.2x1.2x5 | 1.2x1.2x5 | 1.2x1.2x5 | 1.2x1.2x5 | 1.2x1.2x5 | 1.8x1.8x5 |
| Acquired matrix | 128x128 | 128x128 | 128x128 | 128x128 | 128x128 | 128x128 | 128x128 | 128x128 | 128x128 |
| Number of excitations | 2 | 2 | 2 | 2 | 2 | 2 | 2 | 2 | 2 |
